# Why the mycetoma patients are still neglected

**DOI:** 10.1371/journal.pntd.0010945

**Published:** 2022-12-29

**Authors:** Ahmed Hassan Fahal, Kannan Omer Ahmed, Ali Awadallah Saeed, Abdalla Omer Elkhawad, Sahar Mubarak Bakhiet

**Affiliations:** 1 Mycetoma Research Center, University of Khartoum, Khartoum, Sudan; 2 Department of Clinical Pharmacy and Pharmacy Practice, Faculty of Pharmacy, University of Gezira, Wad Medani, Gezira state, Sudan; 3 Department of Pharmacology, Faculty of Pharmacy, The National University, Khartoum, Sudan; 4 Department of Pharmacology, Faculty of Pharmacy, University of Medical Sciences and Technology, Khartoum, Sudan; 5 Department of Molecular biology, Institute of Endemic Diseases, University of Khartoum, Khartoum, Sudan; Gulu University, UGANDA

In 2012, pharmaceutical companies, donors, endemic countries, and nongovernment organisations met in London and signed the London Declaration on Neglected Tropical Diseases. Together, they committed to controlling, eliminating or eradicating ten diseases by 2020, and improving the lives of over a billion people. Great progress has been made, and the declaration has achieved many of its goals [1]. Mycetoma was recognised as a neglected tropical disease (NTD) by the World Health Assembly in 2016 [2]. Global measures have been initiated to assess the current policies and practices worldwide; international consultation to identify priority areas of work and a Global Mycetoma Working Group were established [3].

The 17 Sustainable Development Goals (SDGs) of the 2030 Agenda for Sustainable Development were adopted by world leaders in 2015. They build on the success of the Millennium Development Goals and aim to go further to end all forms of poverty, calling for action by all countries to promote prosperity while protecting the planet. The third goal is the Good Health and Well-Being [3].

The Sixth International Conference on Mycetoma and the First International Training Workshop on Mycetoma were organised in Khartoum, Sudan, by WHO and the Mycetoma Research Centre. The conference delegates have endorsed a “Call for action” urging the global community to work together with multilateral agencies, partners, research institutions, and pharmaceutical companies to address the devastating consequences of this disease [3].

WHO had launched the Roadmap on NTDs, setting out the next decade of action against NTDs, closely aligned with the 2030 SDGs. The roadmap calls for global action and response to prevent, control, eliminate, or eradicate 20 diseases and disease groups and cross-cutting targets associated with the SDGs.

The Kigali Summits on Neglected Tropical Diseases and Malaria, 2020 and 2022, were convened in Kigali and attended by some leading global health voices, world leaders, leading WHO figures, humanitarians, scientific experts, global influencers, and community champions. The Summits issued Calls to Action on the actions needed to end malaria and NTDs. Many delegates had signed the Kigali Declaration and committed to raising funds to accelerate the WHO/NTD 2030 roadmap progress [4].

Despite all this massive international recognition of the NTDs, mycetoma, which is the most neglected of the neglected diseases, had meagre attention. Still, only a few of the research funding bodies had included mycetoma in their priority funding list, few international research consortia on mycetoma were established, it is not on many countries’ health priority agenda, not a reported disease yet, and no new drug for mycetoma is in the pipeline. Furthermore, there is no mycetoma preventive or control measurement or programme yet, and early case detection and management are the only tools to reduce the disease burden. There is no point-of-care diagnostic test for mycetoma hence the late presentation of most of the affected patients [5].

At present, in the area of NTDs, there is an innovation gap, a lack of investment in research and development (R&D), and the traditional challenges involved in the drug discovery process are still existing. Between 2000 and 2011, among 756 new drugs approved, only four new chemical entities (NCEs) were identified for the treatment of malaria, while none were developed against NTDs or tuberculosis. Only 1.4% of approximately 150,000 clinical trials were registered for neglected diseases, with a smaller number of trials for NCEs [6].

For most of the NTDs and mycetoma in particular, the current treatment options are ineffective or have many side effects that limit their use. Following the trend of “big data” in the last few decades, the amount of chemogenomics data has increased significantly. Despite the small amount of NTD data compared to “high-income country” illnesses, the application of computer-aided drug design (CADD) using data available from public repositories affects millions of people and helps accelerate the discovery of potentially life-threatening new treatments. Many NCEs can be designed and discovered by the recent advances in the application of in silico methods, including structure-based and ligand-based drug design, virtual demonstration, quantitative structure–activity relationship, and machine learning, as they can accelerate NCEs discovery [7].

To date, there is only one drug for eumycetoma treatment: itraconazole. Despite the good in vitro activity of voriconazole and posaconazole, their uses in clinical practice are limited because of the longer treatment duration, their high cost, and the relative lack of studies regarding their response. Fosravuconazole, a prodrug of ravuconazole, is the only drug currently in a clinical trial for eumycetoma, comparing its efficacy to itraconazole. It is the first double-blinded, randomized clinical trial (NCT03086226) sponsored by the Drugs for Neglected Diseases Initiative (DNDi), carried out at the Mycetoma Research Center, Sudan, in collaboration with Eisai. Great expectations are placed on fosravuconazole based on its lower side effects and favourable pharmacokinetic profile [8]. Postoperatively, the application of amphotericin B-impregnated absorbable calcium sulfate beads (Stimulan) into the bony defects showed good clinical outcomes with lesser side effects [9].

A recent study showed novel agents with different mechanisms of action were tested for skin NTDs [7]. Presently, some specific pathways to block important fungal molecules for the early discovery of future antifungal pipelines, such as Calcineurin, sphingolipid synthesis of small G-binging proteins, Trehalose pathway, the mitogen-activated protein kinase, and the high-osmolarity glycerol, were reported [8].

In general, NTDs and mycetoma need more research and development support to design and produce NCEs for more efficient treatment. Likewise, there is an urgent need for point-of-care tests and vaccines for this neglected disease. That all need good, sound research collaboration between different institutions for capacity building, technology transfer to mycetoma endemic region, and, hence, good sustainable scientific outcome.
